# AduPARE1A and gemcitabine combined treatment trigger synergistic antitumor effects in pancreatic cancer through NF-κB mediated uPAR activation

**DOI:** 10.1186/s12943-015-0413-2

**Published:** 2015-07-31

**Authors:** Maria Victoria Maliandi, Ana Mato-Berciano, Luciano Sobrevals, Gaël Roué, Anabel José, Cristina Fillat

**Affiliations:** Institut d’Investigacions Biomèdiques August Pi i Sunyer-IDIBAPS, Rosselló 149-153, Barcelona, 08036 Spain; Centro de Investigación Biomédica en Red de Enfermedades Raras (CIBERER), Barcelona, Spain

**Keywords:** Oncolytic adenovirus, Gemcitabine, Pancreatic cancer, uPAR, NF-κB

## Abstract

**Background:**

Combined treatment of oncolytic adenoviruses with chemotherapeutic agents is foreseen as a therapeutic option for cancer. Here we have investigated the potential to use gemcitabine in combination with the oncolytic adenovirus AduPARE1A to treat pancreatic cancer and evaluate the underlying mechanism.

**Methods:**

We treated pancreatic cancer cell lines BxPC-3 and PANC-1 with AduPARE1A and gemcitabine individually or in combination and analyzed cell viability, combination index, apoptosis and viral production. We also investigated the effects of the combination on tumor growth and mice survival in two xenograft models. Furthermore, we analyzed uPAR promoter activity from different uPAR-controlled adenovirus and studied NF-κB mediated effects.

**Results:**

Synergistic cell killing from the combination AduPARE1A/Gemcitabine was observed in BxPC-3 and PANC-1 cells. Moreover, the combination treatment produced therapeutic benefits over either individual modality in two mouse models bearing orthotopic tumors, showing reduced tumor progression and significant prolonged mouse survival. Mechanistic studies showed that the synergistic cell death was not due to an increase in viral replication but occurred through an enhancement of apoptotic cell death. Gemcitabine stimulation increased the transcription of uPAR-controlled transgenes through the induction of NF-κB acting on the uPAR promoter. Interestingly, NF-κB gemcitabine-mediated induction of AduPAR adenoviruses interfered with the activation of NF-κB regulated genes, probably as a result of an intracellular competition for NF-κB DNA binding. Consequently, AduPARE1A infection sensitized cells to gemcitabine-induced apoptosis in the combined treatment.

**Conclusions:**

These data highlights the potential of the combination as a treatment modality for pancreatic cancer patients.

**Electronic supplementary material:**

The online version of this article (doi:10.1186/s12943-015-0413-2) contains supplementary material, which is available to authorized users.

## Background

Pancreatic cancer is a particularly devastating disease, ranked as the fourth leading cause of cancer-related death and a 5-year survival rate of only 6 % [1].

Replication-selective oncolytic adenoviruses are designed to replicate and spread into the tumor, resulting in cancer cell lysis. Oncolytic mutants are being evaluated in the clinic and emerge as a novel approach to treat cancer. Clinical trials in pancreatic cancer patients with ONYX-015, a p53 binding defective oncolytic adenovirus, proved that the administration was safe, although clinical efficacy was not observed when administered alone and only minor responses and stable disease were observed in combination with gemcitabine [2].

We previously developed an oncolytic adenovirus that drives the E1A gene under the control of the urokinase-type plasminogen activator receptor (uPAR) promoter, designated AduPARE1A, and showed its selective replication and its strong antitumor activity in pancreatic cancer models [3, 4]. Despite its anticancer activity demonstrated in the preclinical studies as a monotherapy, multimodal strategies to enhance antitumor efficacy are essential for successful clinical outcome.

Gemcitabine has been the standard chemotherapy treatment in patients with pancreatic cancer since 1997 with modest improvements in median overall survival [5]. More recently, a 2-months increase in overall survival has been observed in the combination treatment of gemcitabine and nab-paclitaxel [6]. Thus most likely patients that will enter clinical trials with oncolytic viruses are expected to be receiving gemcitabine. Combination treatment of oncolytic mutants and gemcitabine has already been explored for given viruses and improved anticancer effects have been reported [7–10]. However, the mechanism of action supporting the benefits of the combination have been quite different in line with the individual characteristics that every oncolytic adenovirus possesses. In fact, a major concern on the combination therapy is that the combination may impede the therapeutic function of each other.

In the current work we have examined the effects of the combination treatment AduPARE1A and gemcitabine, in pancreatic cancer *in vitro* and *in vivo*. We have observed a synergistic anticancer effect *in vitro* and a significant potentiation of tumor growth suppression in xenograft models. Mechanistic studies identified a NF-κB uPAR promoter activation mediated by gemcitabine induction. As a consequence there was increased sensitization to gemcitabine-induced apoptosis in the combined treatment.

## Results

### Combination therapy of gemcitabine and AduPARE1A show synergistic effects

**Fig. 1:**
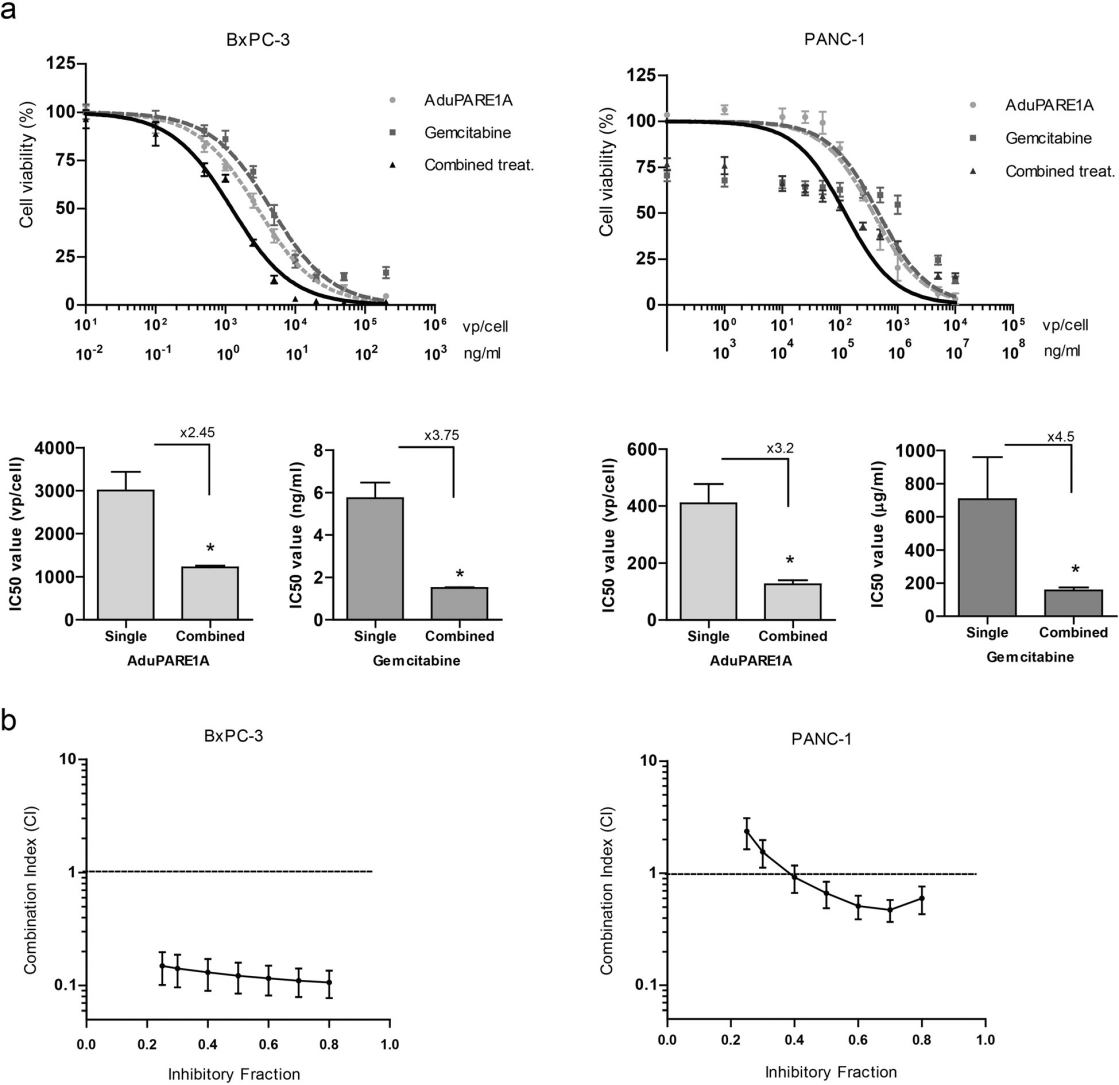
Evaluation of the cytotoxic effects of the combination AduPARE1A plus gemcitabine in cellular models. **a** Dose–response curves of gemcitabine, AduPARE1A or combined treatment in BxPC-3 and PANC-1 cell lines. Cells were seeded in triplicate in a 96-well plate (3 × 10^3^ cells/well) and treated with a dose range of gemcitabine (ng/mL) and/or AduPARE1A (vp/mL). Cell viability was measured 3 days later, and is expressed as the percentage of absorbance of treated cells compared with mock-treated cultures. IC50 values for monotherapy or combination therapy for each treatment are represented in bar graphs. **b** Combination Index values (CI) for the interaction of gemcitabine and AduPARE1A treatments are depicted as a function of inhibitory fractions. Results are expressed as a mean of at least four independent experiments ± SEM

To investigate the potential interest of gemcitabine and AduPARE1A combination therapy for pancreatic cancer, we first examined the cytotoxic effects of individual treatments and their combination in BxPC-3 and PANC-1 cell lines. Cells were infected with different viral doses of AduPARE1A and exposed to several gemcitabine concentrations, alone or in combination, and cell viability of the culture was measured 3 days later. MTT assays showed a dose–response effect in all cases. Combination treatment significantly reduced the IC50 of each agent, in both BxPC-3 and PANC-1 cells (Fig. 1a).

Pharmacological interaction between the above-mentioned treatments was assessed by Combination Index analysis (CI) in both PANC-1 and BxPC-3 cells. CI was calculated for 20–80 % fractional inhibition. In Bx-PC-3, at all the fractions analyzed, the CI was lower than one, indicating a potentiation effect of AduPARE1A when combined with gemcitabine, and vice versa, highlighting that both treatments were synergistic. In PANC-1 cells synergism started to show at 40 % of the inhibitory fraction (Fig. 1b).

### In vivo multimodal gemcitabine and AduPARE1A therapy significantly improved mouse survival over either individual therapy in animals bearing orthotopic BxPC-3 and PANC-1 tumors

**Fig. 2:**
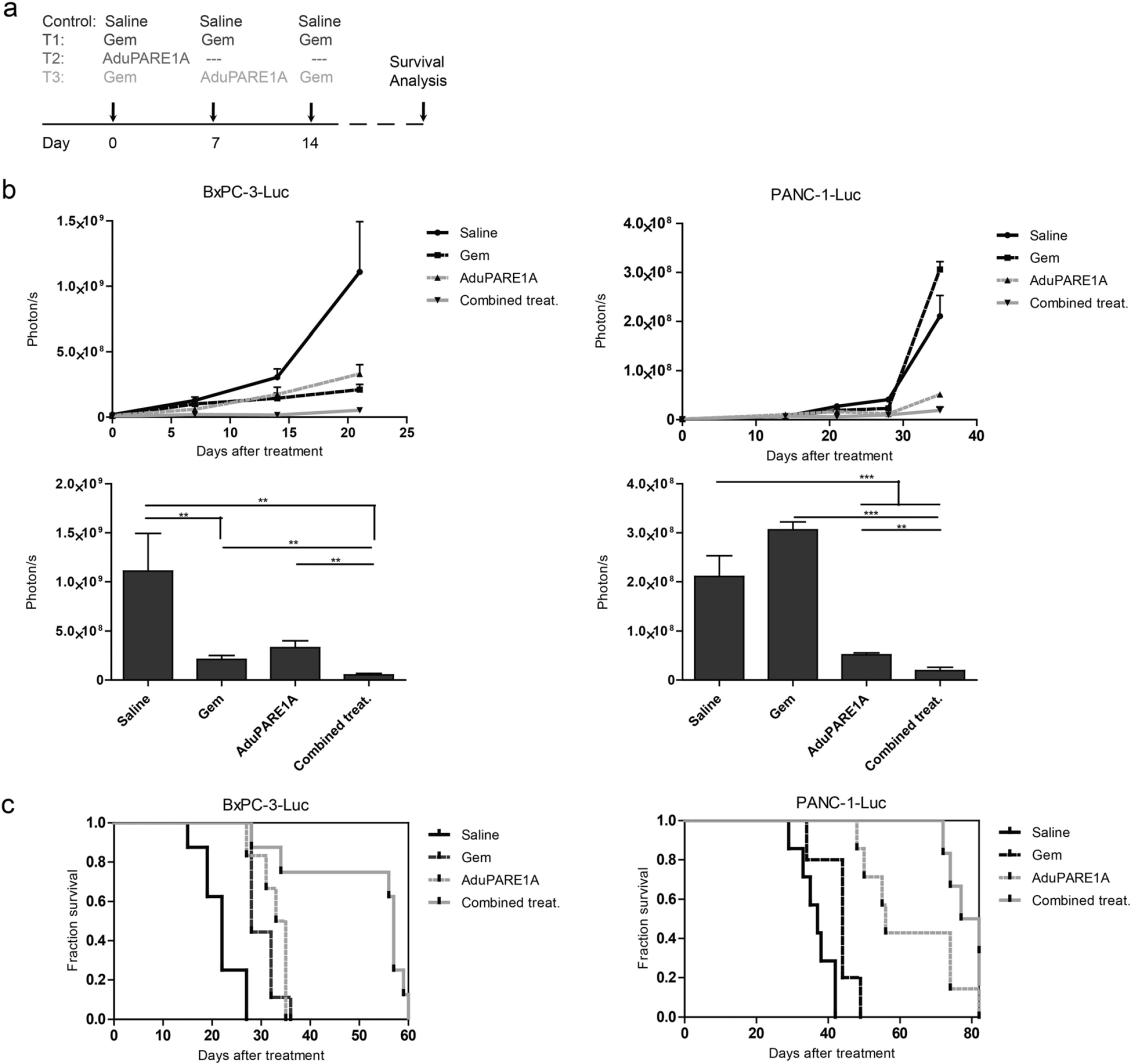
Antitumoral activity of combined AduPARE1A plus gemcitabine treatment in BxPC-3-Luc and PANC-1-Luc orthotopic tumors. **a** Schematic representation of treatment protocols applied in mice bearing BxPC-3 and PANC-1 orthotopic tumors. BxPC-3-Luc and PANC-1-Luc orthotopic tumors were treated with a weekly dose of 160 mg/kg of gemcitabine for 3 weeks (T1), with a single dose of AduPARE1A (5 × 10^10^ vp) (T2), with the combined treatment, replacing a dose of gemcitabine for the AduPARE1A (T3), or with saline in the control group. **b** Luciferase quantification of bioluminescent emission images from BxPC-3-Luc and PANC-1-Luc tumor-bearing mice, at different time points. Bars graph represent luciferase quantification at 21 and 35 days after treatment in BxPC-3 and PANC-1, respectively. Results are expressed as photons per second. Values are represented as mean ± SEM). (*p < 0.05; **p > 0.01; ***p < 0.001). **c** Kaplan-Meier survival curves in BxPC-3-Luc tumors: (T1: log rank test = 0.003, T2: log rank test = 0.001, T3: log rank test = 0.0001); in PANC-1-Luc tumors (T1: log rank test = 0.014, T2: log rank test = 0.0002, T3: log rank test = 0.0006). BxPC-3-Luc xenografts: saline: *n* = 8; Gem: *n* = 9; AduPARE1A *n* = 6; combined: *n* = 8. PANC-1-Luc xenografts: saline: *n* = 7; Gem: *n* = 6; AduPARE1A *n* = 7; combined *n* = 6

To analyze the impact of the combined treatment *in vivo*, BxPC-3-Luc and PANC-1-Luc mice bearing orthotopic tumors were treated with intravenous injection of a single dose of AduPARE1A 5 × 10^10^ (vp/mouse), 3 weekly doses of gemcitabine (160 mg/Kg) or a combination treatment based on a weekly dose of gemcitabine and a single AduPARE1A dose, following the protocol indicated in Fig. 2a. All treatments significantly reduced tumor progression, except for gemcitabine treatment in PANC-1 xenografts, as indicated by bioluminescence quantification analysis (Fig. 2b). Mouse survival was significantly prolonged in the combined treatment with respect to gemcitabine or AduPARE1A monotherapy (Fig. 2c). The median survival in mice bearing BxPC-3 Luc tumors was of 22 days for the saline group and of 57 days for the combined treatment. The median survival in mice bearing PANC-1-Luc tumors was of 37 days for the saline treated group and of 79.5 days for the combined treatment. The enhanced anticancer effects correlated with increased cleaved active caspase-3 immunostaining in the combined treatment, suggesting enhanced apoptosis (Additional file 1: Figure S1).

### AduPARE1A and gemcitabine combined treatment decrease viral replication but increase apoptotic cell death

**Fig. 3:**
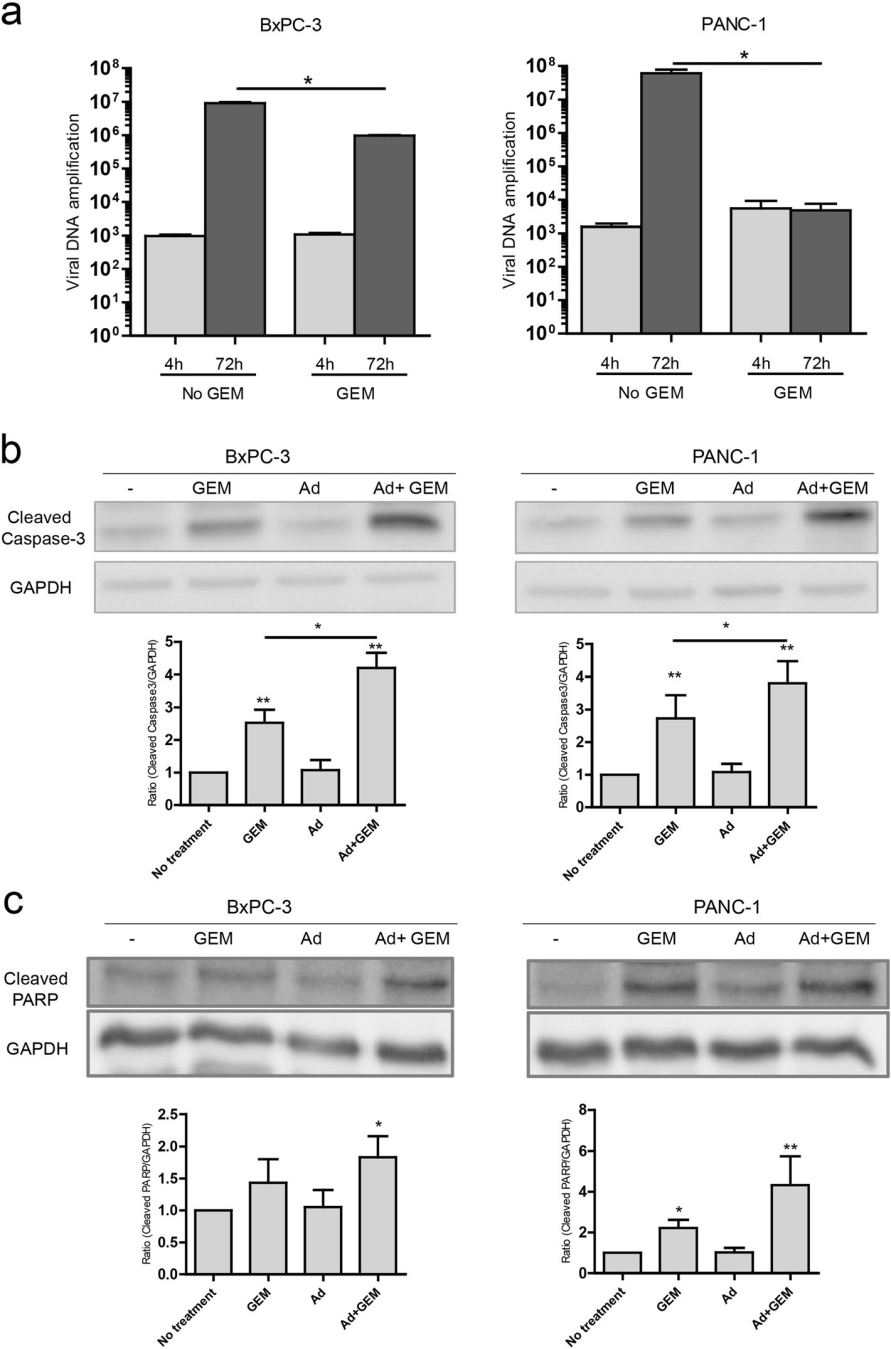
Analysis of the effects of combined treatments on AduPARE1A production and the level of apoptotic cell death in BxPC-3 and PANC-1 cell lines. **a** AduPARE1A viral production in BxPC-3 and PANC-1 cell lines at 72 h after infection. Cell lines were infected with AduPARE1A (4000 vp/cell; 200 vp/cell, BxPC-3 and PANC-1 respectively) and 4 h later the medium was removed and treated, or not, with Gemcitabine (50 ng/ml; 500 μg/ml, BxPC-3 and PANC-1 respectively) for 72 h. Viral DNA obtained from whole cell extracts at 4 and 72 h was analyzed for hexon amplification by qPCR. Values are represented as a mean ± SEM of four independent experiments (*P < 0.05). **b** and **c** Western blot of cleaved caspase-3 (**b**) and cleaved PARP (**c**) in BxPC-3 and PANC-1 cell lines treated with mock, gemcitabine (50 ng/mL; 500 μg/mL, BxPC-3 and PANC-1 respectively), AduPARE1A (4000 vp/cell; 200 vp/cell, BxPC-3 and PANC-1 respectively) or AduPARE1A plus gemcitabine at 48 h after treatment. Bar graphs show quantification of cleaved caspase-3 and cleaved PARP related to GAPDH expression in *n* = 5 (BxPC-3) and *n* = 6 (PANC-1) independent experiments. (*P < 0.05, **P < 0.01)

To understand the cell death mechanisms mediating the effects of the combined treatment, we analyzed whether gemcitabine-induced effects on viral replication could be involved in the increase of cell killing. BxPC-3 and PANC-1 cells were transduced with 4.000 vp/cel or 200 vp/cel of AduPARE1A and treated with 50 ng/ml or 500 μg/ml of gemcitabine respectively. Viral replication was assessed at 4 h after transduction, corresponding to the viral input dose, and at 72 h. In the absence of gemcitabine significant viral replication could be observed in both cell types. However, the presence of gemcitabine slightly impaired adenovirus replication in BxPC-3 cells, and completely blocked it in PANC-1 cells (Fig. 3a). These results exclude the increase of viral replication as a mechanism to enhance cell death in the context of combined treatment. Next, we evaluated the effects of the combined treatment on apoptotic cell death. BxPC-3 and PANC-1 cells were exposed to AduPARE1A and gemcitabine, either alone or in combination. The same viral doses indicated above and gemcitabine concentrations of 50 ng/ml in BxPC-3 or 500 μg/ml in PANC-1 cells were used. Apoptotic cell death was measured 48 h later by the expression of activated caspase-3 and cleaved PARP. Western blot analysis shows that, in both cell lines, the expression of activated caspase-3 was significantly increased in the combination treatment when compared to gemcitabine alone. Cleaved PARP was also significantly increased in the combination treatment. No apoptotic effect was observed with the virus alone (Fig. 3b, c). These results suggest that AduPARE1A sensitizes pancreatic cancer cells to gemcitabine-induced apoptosis.

### Gemcitabine induces the activity of uPAR-controlled adenoviruses

**Fig. 4:**
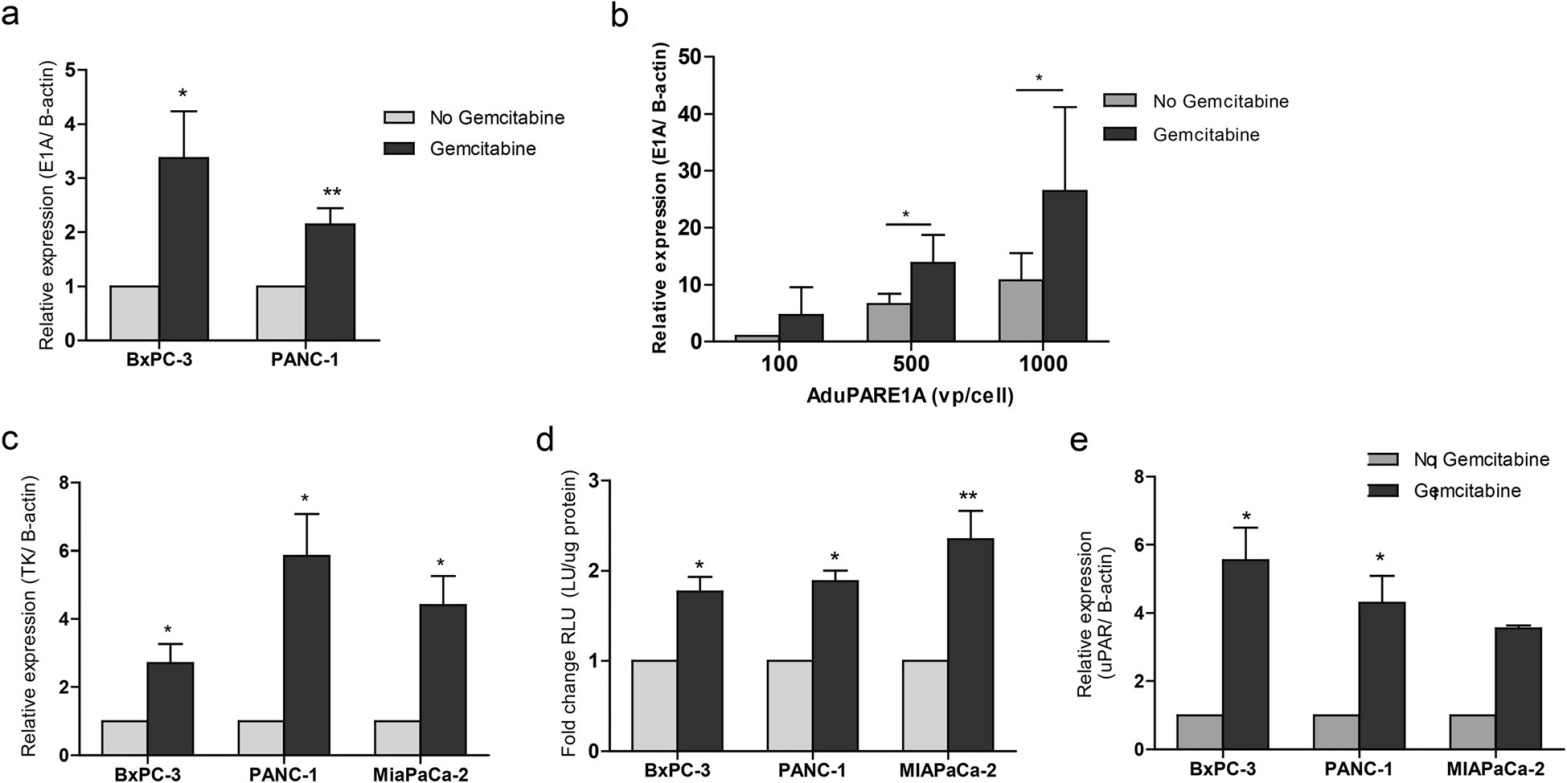
Evaluation of the effects of gemcitabine on the transcriptional activation of the uPAR promoter. **a** Analysis of E1A gene expression in BxPC-3 and PANC-1 cells infected with AduPARE1A and treated with gemcitabine (20 ng/ml and 250 μg/ml) for 24 h. **b** Analysis of E1A gene expression in BxPC-3 cells infected with increasing viral doses in the presence or absence of 10 ng/ml of gemcitabine. **c** Analysis of TK gene expression in BxPC-3, PANC-1 and MIAPaCa-2 cell lines transduced with AduPARTK^T^ (18.790 vp/cell, 1466 vp/cell and 11716 vp/cell, respectively) and treated with gemcitabine (20 ng/ml, 250 μg/ml and 20 ng/ml respectively) for 24 h. **d** Luciferase activity in BxPC-3, PANC-1 and MIAPaCa-2 cell lines transduced with 10 MOI (BxPC-3, PANC-1) or 20 MOI (MIAPaCa-2) of AduPARLUC and treated or not with gemcitabine (20 ng/ml, 250 μg/ml and 20 ng/ml respectively) for 24 h. Results are expressed as RLU (LU/μg of protein). **e** Expression of endogenous uPAR gene in BxPC-3, PANC-1 and MIAPaCa-2 cell lines treated with gemcitabine (20 ng/ml, 250 μg/ml and 20 ng/ml respectively) for 24 h. Results are represented as the mean ± SEM of at least three independent experiments

To investigate the mechanism involved in the synergistic anti-cancer effects of AduPARE1A and gemcitabine we first evaluated the expression of the E1A viral gene in BxPC-3 and PANC-1 cultures infected with AduPARE1A in the presence or absence of gemcitabine. We observed a significant increase in the E1A mRNA transcript in the presence of gemcitabine in the two cells lines analyzed (Fig. 4a). E1A induction in gene expression by gemcitabine was observed at escalating viral doses (Fig. 4b).

To evaluate whether the increase in E1A expression was the consequence of uPAR promoter activation mediated by gemcitabine stimuli, we decide to analyze the effects of gemcitabine on the expression of different transgenes also controlled by the uPAR promoter. BxcPC-3, PANC-1 and MIAPaCa-2 cells were transduced with AduPARTK^T^, a non-replicative adenovirus that expresses a modified form of the Herpes simplex thymidine kinase gene under the control of the uPAR promoter, in the presence or not of gemcitabine. Analysis of TK expression revealed increased levels of TK mRNA in the presence of gemcitabine (Fig. 4c). Cell transduction with AduPARLuc, a reporter adenovirus that contains the luciferase expression regulated by the uPAR promoter, showed that luciferase activity was significantly higher in cells that received AduPARLuc and gemcitabine (Fig. 4d). Furthermore, gemcitabine treatment also induced endogenous uPAR mRNA overexpression (Fig. 4e).

These findings indicate that gemcitabine enhances uPAR promoter transcriptional activity.

### NF-κB activation mediates gemcitabine uPAR-induction

**Fig. 5:**
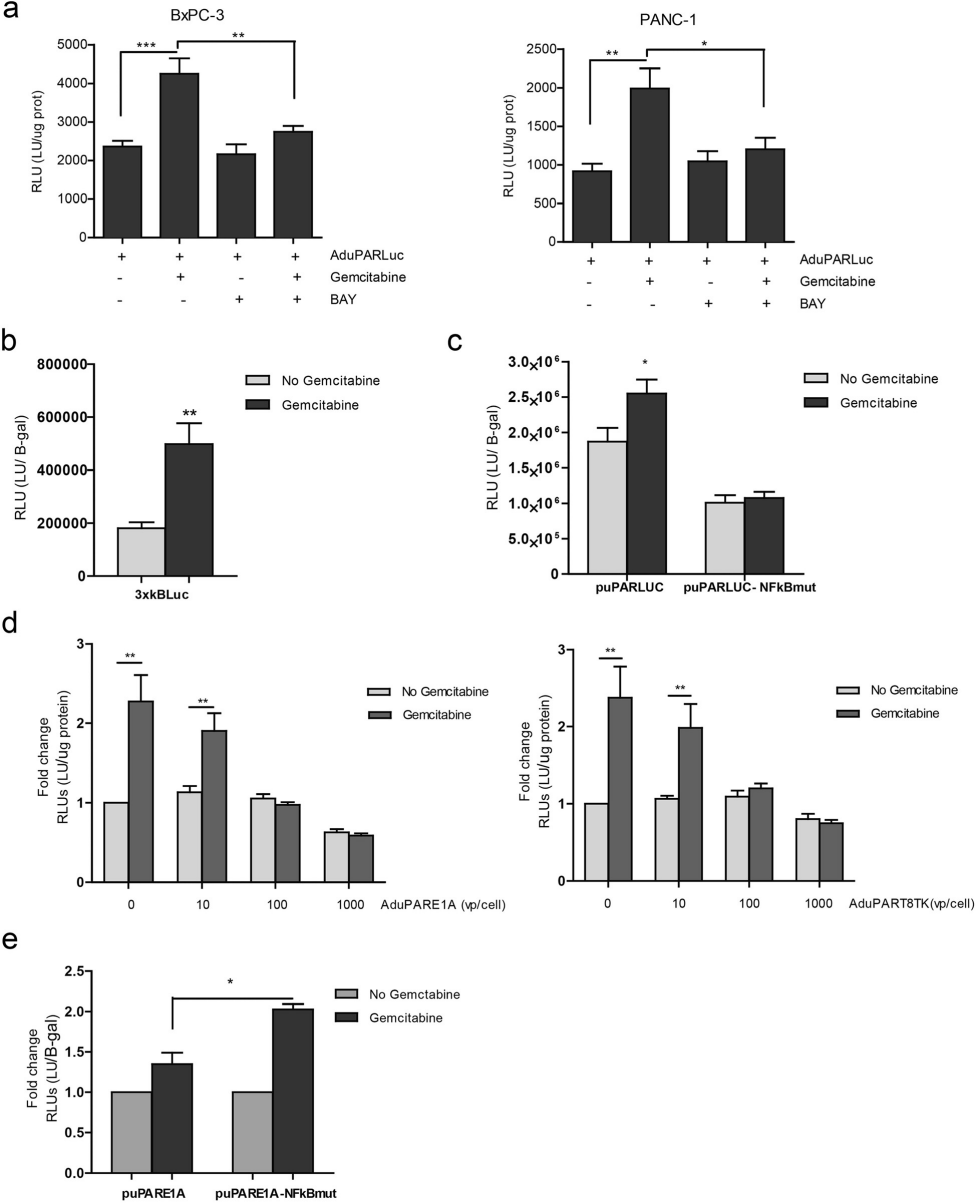
Gemcitabine promotes the activity of NFkB, leading to an increase in uPAR promoter activation. **a** Luciferase activity in BxPC-3 and PANC-1 cell lines transduced with AduPARLuc, 10 or 20 MOI respectively, and treated with 5 μM of kinase inhibitor BAY 11-7082 (Calbiochem) for 1 h, and then exposed to (20 ng/ml and 250 μg/ml) of gemcitabine for 24 h. Results are expressed as light units (LU) normalized to total protein levels (RLU). **b** Luciferase activity of 3xκB-Luc reporter plasmid in 293T cells treated with 200 ng/ml of gemcitabine for 24 h. Results are expressed as LU normalized to β-gal activity. **c** Luciferase activity of puPARLuc and puPAR-NFκBmut reporter plasmid in 293T cells treated with 200 ng/ml gemcitabine for 24 h. Results are expressed as LU normalized to β-gal activity. **d** Luciferase activity of 3xκB-Luc reporter plasmid in 293T cells transduced with increasing doses of AduPARE1A (*left panel*) or AduPARTK^T^ (*right panel*) and treated or not with 200 ng/ml gemcitabine. Results are expressed as fold-change of LU normalized to total protein levels (RLU). Values represent the mean ± SEM of five independent experiments. **e** Luciferase activity of 3xκB-Luc reporter plasmid in 293T cells co-transfected with puPARE1A or puPARE1A-NF-κBmut, in the presence or absence of 200 ng/ml gemcitabine. Results are expressed as fold-change of LU normalized to β-gal activity (RLU). Values represent the mean ± SEM of four independent experiments

Next we asked how gemcitabine could mediate the activation of uPAR-controlled transgenes. Gemcitabine treatment has been shown to induce NF-κB activation in small cell lung cancer and pancreatic cancer cells [11, 12]. The proximal uPAR promoter fragment of 400 bp, used in the uPAR-adenoviruses, contains an NF-κB-like site at position −45 that specifically binds the transcription factor NF-κB, upregulating the uPAR gene [13]. To study the involvement of NF-κB mediating uPAR activation by gemcitabine in AduPAR-regulated viruses, PANC-1 and BxPC-3 cells were transduced with AduPARLuc and treated or not with gemcitabine for 24 h in the presence or absence of BAY 11-7082 (BAY), an inhibitor of κB kinase (IKK). As shown in Fig. 5a, uPAR promoter activity was induced by gemcitabine, and such induction was strongly suppressed by the BAY inhibitor in both PANC-1 and BxPC-3 cells. The gemcitabine stimuli triggering NF-κB activation was also evident in the NF-κB reporter transfection experiments (Fig. 5b). To further demonstrate the involvement of NF-κB mediating gemcitabine-induced uPAR activation, cells were transfected with puPARLuc or a plasmid with NF-κB mutagenized sites in the uPAR promoter, the puPARLuc-NF-κB mut. The mutation of the NF-κB binding site in the uPAR promoter abrogated the gemcitabine induced uPAR activation observed in puPARLuc transfected cultures (Fig. 5c). The above results strongly support the involvement of NF-κB in the gemcitabine-mediated uPAR activation. However, NF-κB is thought to play an antiapoptotic role and NF-κB signaling pathways are often regarded as mediators of chemoresistance [14]. Next we aimed to understand how the increase uPAR promoter activation by NF-κB- gemcitabine induction could mediate the synergistic cell killing effect in the AduPARE1A and gemcitabine combined treatment. To this end, cells were transduced with increasing doses of AduPARE1A in the presence or not of gemcitabine and transfected with the 3xκB-Luc reporter plasmid. Increased concentration of viral particles strongly suppressed the gemcitabine induction of the NF-κB responsive plasmid, while E1A was transcriptionally activated (Fig. 5d, Additional file 2: Figure S2). Similar results were observed with the AduPARTK^T^ (Fig. 5d), suggesting that the effect was independent of E1A, and probably the large amount of viral particles in the cells exerted a decoy effect of NF-κB, interfering with its natural binding to endogenous NF-κB responsive genes. Interestingly, gemcitabine induction of the NF-κB responsive plasmid was superior in cells expressing E1A from a uPAR promoter with NF-κB mutagenized sites (puPARE1A- NF-κBmut) than from its wild-type form, suggesting that in a context in which NF-κB can not bind to the uPAR promoter there is no decoy effect and NF-κB-response genes could be activated (Fig. 5e). These data further supports an E1A-independent effect since E1A will be poorly expressed in the gemcitabine treated puPARE1A-NF-κBmut cultures, because this mutation abrogates gemcitabine activation of the promoter as shown in Fig. 5c.

Collectively, these results suggest that higher amounts of AduPARE1A viral particles will interfere with NF-κB activation, facilitating gemcitabine-induced apoptosis, leading to *in vitro* synergistic cell death and improve antitumor response of the combination treatment.

## Discussion

Oncolytic adenoviruses are now under evaluation in clinical trials in patients with pancreatic cancer [15–17]. These agents are attractive treatment options either alone or in combination with traditional cytotoxic drugs. Previous work from our group has shown the antitumor efficacy and tumor selectivity of AduPARE1A [3, 4]. The current study aimed to determine the efficacy to combine AduPARE1A and gemcitabine commonly used to treat pancreatic cancer. We found a synergistic cell killing effect in the combination treatment. *In vivo*, in two orthotopic xenograft models, combined treatment prolonged mouse survival compared to either therapy alone providing evidence of the efficacy of AduPARE1A plus gemcitabine for pancreatic cancer. Furthermore, we provide evidences on the mechanisms of such synergistic interaction.

The increased cell death in the combined treatment was not due to an improvement in viral replication, on the contrary, replication was attenuated by the presence of gemcitabine. The effect was more pronounced in PANC-1 cells, where much higher doses of gemcitabine were used. In fact, viral attenuation by DNA damaging agents has also been observed in the E1B19K oncolytic adenoviral mutants [8] and in replication-selective herpes mutants targeting pancreatic cancer cells [18]. The G1/S cell-cycle block triggered by gemcitabine limits DNA cellular synthesis, hampering to support viral propagation [8].

Interestingly, the increased cell death in the combination treatment could be explained by an enhancement of gemcitabine-induced apoptosis since we observed an augmented activity of caspase-3. Several authors have postulated that increased E1A expression might be responsible for most of the synergistic effects in combination treatments of oncolytic adenovirus and DNA damaging agents, since E1A is a potent apoptotic inducer [8, 10, 19, 20]. The pro-apoptotic functions of E1A, have been linked to its capacity to upregulate Egr-1 which is directly involved in Bim transactivation. The alterations of Bim contribute to Bax activation and subsequent caspase activation [21]. We observed an increase in E1A expression upon gemcitabine treatment, suggesting that at least part of the synergism could rely on the pro-apoptotic functions of E1A. However this seems unlikely since AduPARE1A oncolytic adenovirus retains all the viral functions and infected cells also express the anti-apoptotic E1B and E3 viral proteins, what may hamper or at least mitigate E1A activity. In fact, AduPARE1A alone was not able to trigger caspase-3 activation. Thus, other mechanisms might be operating in the AduPARE1A cooperation to gemcitabine-induced apoptosis. It should be considered that E1A has been shown to induce mesenchymal-to epithelial conversion resulting in the formation of gap junctions, what facilitates the cell-to cell transfer of gemcitabine toxic metabolites and the spread of gemcitabine cytotoxicity [22, 23].

We observed that the increase in E1A expression by the combined treatment was the consequence of a transcriptional activation of the uPAR promoter mediated by gemcitabine. Induction of transgene expression was extended to all uPAR-promoter controlled adenoviral vectors analyzed such as the AduPARTK^T^, increasing TK transcriptional levels and the AduPARLuc, enhancing luciferase activity. Furthermore, gemcitabine induced transcriptional activation of the endogenous uPAR gene. These data highlight that gemcitabine induction of uPAR adenoviruses enhances the biological effects of the controlled transgenes. Such increase in transgene expression could partially account by the gemcitabine enhancement of viral infectivity, as reported by some authors [10, 24]. However, since we observed a gemcitabine-mediated increase in the uPAR promoter transcriptional activity, most likely the increased transgene expression will result from the interaction of different transcription factors to cis acting regulatory sequences in the uPAR gene [25]. One of the key factors regulating the uPAR promoter is NF-κB [13]. NF-κB is constitutively active in pancreatic cancer and gemcitabine is able to induce NF-κB binding activity. In this line, we observed that gemcitabine induced-uPAR promoter activation was completely blocked by the NF-κB inhibitor BAY11-7082, and gemcitabine was unable to activate a uPAR promoter with NF-κB mutagenized binding sites. However, activation of NF-κB has been linked to reduced cytotoxicity and antiapoptotic effects, and it is proposed as a mechanism of gemcitabine resistance [26]. Interestingly, what we observed is that in the combination treatment of AduPARE1A and gemcitabine, at high viral doses, where E1A is induced, AduPARE1A interferes with the activation of NF-κB regulated genes. E1A has been proposed to counteract gemcitabine induced NF-κB activation sensitizing cells to apoptosis [27], however in our studies the suppression of NF-κB activation would not only account by E1A since the same kind of interference was observed with AduPARTK^T^. These results support the notion that there will be an intracellular competition for NF-κB DNA binding, favoring AduPAR promoter activation. As a consequence E1A will be highly expressed. It could be speculated that in this scenario endogenous NF-κB- responsive antiapoptotic genes might be suppressed leading to reversal of gemcitabine resistance and enhancement of apoptosis. The proposed mechanism of synergistic activity between AduPARE1A and gemcitabine is a novel mechanism that could also extent to other viruses susceptible to NF-κB regulation. Furthermore, the remarkable *in vivo* anti-cancer effects could also result from the capacity of oncolytic adenovirus to disrupt tumor architecture thus facilitating gemcitabine penetration [28].

## Conclusions

In summary in the current study we show that the combination of AuPARE1A and gemcitabine has a synergistic cell killing effect that holds potential as a novel therapy for pancreatic cancer treatment. We propose a model of the operating mechanism underlying the synergistic effects, through which gemcitabine will trigger an NF-κB activation to induce uPAR promoter activity, leading to an increase in E1A protein. The NF-κB transcription factor binding to the high number of uPAR sequences in the adenovirus will produce a sponge effect, interfering with NF-κB activation of antiapoptotic genes, thus sensitizing gemcitabine-induced apoptosis.

## Methods

### Cell lines

Human pancreatic tumor cell lines: BxPC-3, PANC-1 and MIAPaCa-2, and 293T cells were obtained from the American Type Culture Collection (ATCC; Rockville, MD, USA). Luciferase-expressing cells PANC-1-Luc and BxPC-3-Luc were established as previously described [3, 29]. Cell lines were maintained in Dulbecco’s modified Eagle’s medium (DMEM), supplemented with 10 % fetal bovine serum (FBS), penicillin (100 U/ ml), streptomycin (100 μg/ ml), and glutamine (2 mM) (GIBCO-Invitrogen). Every 2 months cells were plated from a frozen vial of the original batch but were not authenticated by the authors. Interspecies contamination was tested by PCR routinely.

### Chemicals and antibodies

Gemcitabine (Hospira UK Limited) was dissolved in water to a concentration of 10 mg/ml. BAY 11-7082 (Calbiochem, La Jolla, CA) was dissolved in dimethyl sulfoxide, stored at −20 °C, and protected from light. The following antibodies were used in western blot analysis: Rabbit anti-Cleaved Caspase-3 (Asp175) (Ref.9661, Cell Signaling Technology, MA, USA), Rabbit anti-PARP (Cat. No.11835238001, Roche), Rabbit anti-GAPDH (Ref.ABS16, Merck Millipore, Germany), HRP-conjugated Goat anti-Rabbit (DakoCytomation, Denmark).

### Adenoviruses

Replication-defective adenoviruses AduPARLuc express the firefly luciferase gene under the control of uPAR promoter. This virus has already been described [3]. The oncolytic AduPARE1A was constructed by inserting the uPAR promoter (450-bp fragment) upstream the E1A adenoviral gene [3]. AduPARTK^T^ encodes a modified Herpes Simplex virus thymidine kinase gene, as described [30]. Experiments with AduPARE1A and AduPARTK^T^ were conducted with CsCl purified viral preparations and results are expressed as vp/ml. AduPARLuc experiments were performed with homogenates from viral infected cells subjected to three rounds of freeze-thaw lysis and viral doses are expressed as MOI.

### Plasmid constructs

A 450-bp fragment of the uPAR promoter ( −402/+48 region) and the luciferase gene present in the pAd-TrackuPARLuc [3] were excised and cloned into a pGEM-T vector (puPARLuc). The reporter plasmid puPARLuc-NF-κBmut was generated by directed mutagenesis. The NF-kB binding site in the uPAR promoter (located at −45 bp of transcription initiation site) 5'-GGGAGGAGT-3' [13], was mutated to 5'-GGATCCAGT-3', using the QuikChange Multi Site-Directed Mutagenesis Kit (Agilent, CA USA), as recommended by the manufacturer and using the mutagenesis primer: 5'-ctctttcgcaaaacgtctggatccagtccctggggccacaaaac-3'. puPARE1A was generated by cloning the uPARE1A construct in a pGEM-T vector. The NF-κBmutation in the puPARE1A to generate puPARE1A-NF-κBmut was carried out by directed mutagenesis as described above. 3xκB-Luc reporter plasmid was previously described [31].

### Reporter gene assays

With Adenoviruses: Cells were transduced with AduPARLuc, 4 h later virus was removed and replaced with fresh medium containing or not BAY 11-7082 (Calbiochem) for 1 h and then exposed to gemcitabine for 24 h. Luciferase activity was measured in a luminometer (Synergy HT, Biotek) with luciferin as a substrate (Promega, Madison,USA). Results are expressed as light units (LU) and normalized to total protein levels.

With Reporter plasmids: 293T cells were seeded (40.000 cells/well) and co-transfected with luciferase reporter plasmids (150 ng/well): 3xκB-Luc, puPARLuc or puPARLuc-NF-κBmut or puPARE1A or puPARE1A-NF-κBmut and the β-gal reporter plasmid (30 ng/well). CalPhos mammalian transfection kit (Clontech, Takara Bio Company Inc.) was used to transfection, following the manufacturer guidelines. At 16 h post-transfection the medium was change and new fresh medium or medium containing 200 ng/ml of Gemcitabine was added. Twenty-four hours later, cell lysates were analyzed for luciferase activity using Luciferase Assay System (Promega), and β-galactosidase activity was used as a control of transfection efficiency.

### Dose–response analysis and combination index analysis

BxPC-3 and PANC-1 cell lines were seeded in 96-well plates at a density of 3 × 10^3^ cells per well. Cells were treated with different doses of gemcitabine (ng/mL), AduPARE1A (vp/cell) or the combination of both treatments, maintaining a constant ratio of gemcitabine:adenovirus of 1:800 or 1250:1 in BxPC-3 and PANC-1 respectively. Cell viability was measured 3 days later, using the MTT colorimetric assay (Ref.19265 USB, Affymetrix, CA USA). The induction of synergism, summation or antagonism between gemcitabine and AduPARE1A treatments was analyzed by Combination Index analysis, by the adapted method of Chou and Talay [32, 33]. Dose–response curves were obtained for combined or single treatments by a standard non-linear regression using GraphPad Prism Software (CA, USA), and IC50 and combination index values were calculated using Calcusyn Software (Biosoft, Cambridge UK). CI >1 indicates synergism, *CI* = 1 additivity and CI <1 indicates antagonism between treatments.

### Quantitative RT-PCR analysis of gene expression

Total RNA was extracted from different cells lines using the RNeasy® Mini Kit (Qiagen). The cDNA was synthesized from total RNA using a reverse transcriptase and random hexamers (RETROscript® Kit, Ambion), according to the manufacturer’s instructions. The PCR reactions were performed in a total volume of 10 μl containing SYBR Green I Master Mix (Roche) with 10 μM of each primer and 1 μl cDNA. Quantitative PCR was performed in triplicate with the ViiA™ 7 System (Applied Biosystems). Primers sequences for the different genes are listed in Additional file 3: Table S1.

### Western blot analysis

Cell lines were treated for 48 h with gemcitabine or/and infected with AduPARE1A. After treatment, floating and adherent cells were collected in lysis buffer (62.5 mM Tris HCl pH6.8, 2 % Sodium Dodecyl Sulfate and 10 % Glicerol) containing 1 % Complete Mini-Protease Inhibitor Cocktail (Roche Diagnosis, Switzerland), and 5 mM NaF. Cell lysates were boiled for 10 min at 98 °C. All protein extracts were quantified using BCA kit (Thermo scientific, MA USA) and 80 μg of protein was resolved in 12 % SDS-PAGE and transferred to PVDF or nitrocellulose membrane (Merk Millipore, Germany). Membranes were immunobloted with anti-cleaved caspase-3 (1/200, overnight at 4 °C) or anti-PARP (1/1000, 2 h at room temperature) and anti GAPDH (1/3000, 1 h at room temperature), rinsed with TBS-Tween and then incubated with HRP-conjugated goat anti rabbit (1/2000, 1 h at room temperature). Antibody labeling was detected by Enhanced Chemoluminiscence Kit (Amersham, GE Healthcare, Switzerland).

### Viral genomes quantification

BxPC-3 and PANC-1 cells were infected with 4000 vp/cell and 200 vp/cell, respectively. Culture medium was removed at 4 h after infection and cells were treated, or not with 50 ng/ml and 500 μg/ml of gemcitabine (in BxPC-3 and PANC-1 respectively). Viral DNA was obtained from cell and supernatant (lysed by three freeze and thaw cycles) at 4 and 72 h after infection, using the UltraClean BloodSpin DNA Isolation Kit (Mo Bio Laboratories, CA USA) according to manufacturer’s instructions. Viral genomes were determined by Real Time PCR using SYBR Green I Master mix (Roche Diagnostics, Switzerland) and Hexon primers (described in Additional file 3: Table S1). The adenovirus copy number was interpolated in a standard curve, consisting of adenoviral DNA dilutions in a background of genomic DNA.

### Bioluminescence assay and quantification

Animals were anesthetized with a mixture of isofluorane and oxygen preparation and the substrate d-Firefly-Luciferin (Xenogen, Alameda, CA) was administered i.p (32 mg/kg). Luciferase activity was visualized and quantified using an *in vivo* bioluminescent system (IVIS50; Xenogen) and Living Image 2.20.1 Software overlay on Igor Pro4.06A software (Wavematrics, Seattle, WA) was used, as previously described [3]. Luciferase activity was quantified from non-saturated images, measuring the total amount of emitted light recorded by the CCD camera.

### Antitumoral efficacy

Male athymic *nu/*nu mice (6–8 weeks old, Harlan Iberica) were used to generate human orthotopic pancreatic tumors. Animal procedures met the guidelines of European Community Directive 86/609/EEC and were approved by the Local Ethical Committee.

Orthotopic human pancreatic cancer xenografts were generated as previously described [29]. Briefly, 5 × 10^5^ PANC-1-Luc and BxPC-3-Luc cells were injected into the pancreas of 8-week-old male athymic nude mice, in a final volume of 50 μl. When tumors reached 10^6^–10^7^ photons/s measured by *in vivo* bioluminescence, which corresponds to approximately 100 mm^3^, animals were randomly divided into four groups: Saline, GE, AduPARE1A and AduPARE1A + GE, and were i.v injected with AduPARE1A 5 × 10^10^ vp or PBS and or gemcitabine 160 mg/Kg or PBS following the protocol described in Fig. 2a. Tumor growth was monitored by bioluminescence analysis. Survival studies were performed and animals were sacrificed according to ethical guidelines.

### Statistical analysis

Results are expressed as mean ± SEM of at least three independent experiments. Statistical differences were determined using Prism (version 5; GraphPad software) and were considered significant for *P* values less than 0.05. Survival studies were performed to analyze time-to-event probability using the SPSS software. The survival curves (Kaplan–Meier curves) obtained were compared for the different treatments. A log-rank test was used to determine the statistical significance of the differences in time-to-event. A *p* value of less than 0.05 was considered statistically significant.

## Additional files

Additional file 1: Figure S1. A) Schematic representation of treatment protocols applied in mice bearing BxPC-3 subcutaneous tumors. B) Representative images of IHC staining for Cleaved-Caspase3 (Ref.9661, Cell Signaling Technology) in BxPC-3 tumors. Bar graph shows quantification of cleaved-caspase3 cells per field. Results are represented as the mean ± SEM of n ≥6 fields. (JPEG 110 kb) Additional file 2: Figure S2. Expression of E1A gene in 293T cells infected with AduPARE1A (1000 vp/cell) and treated, or not, with gemcitabine (200 ng/ml). Results represented are the mean ± SEM of at least four independent experiments. (DOCX 40 kb) Additional file 3: Table S1. Primer sequences. (JPEG 1145 kb)
