# Supplementary figures and images for: AduPARE1A and gemcitabine combined treatment trigger synergistic antitumor effects in pancreatic cancer through NF-κB mediated uPAR activation
Source: Mol Cancer. 2015 Jul 31;14:146. doi: 10.1186/s12943-015-0413-2 (PMC4521493; doi:10.1186/s12943-015-0413-2)

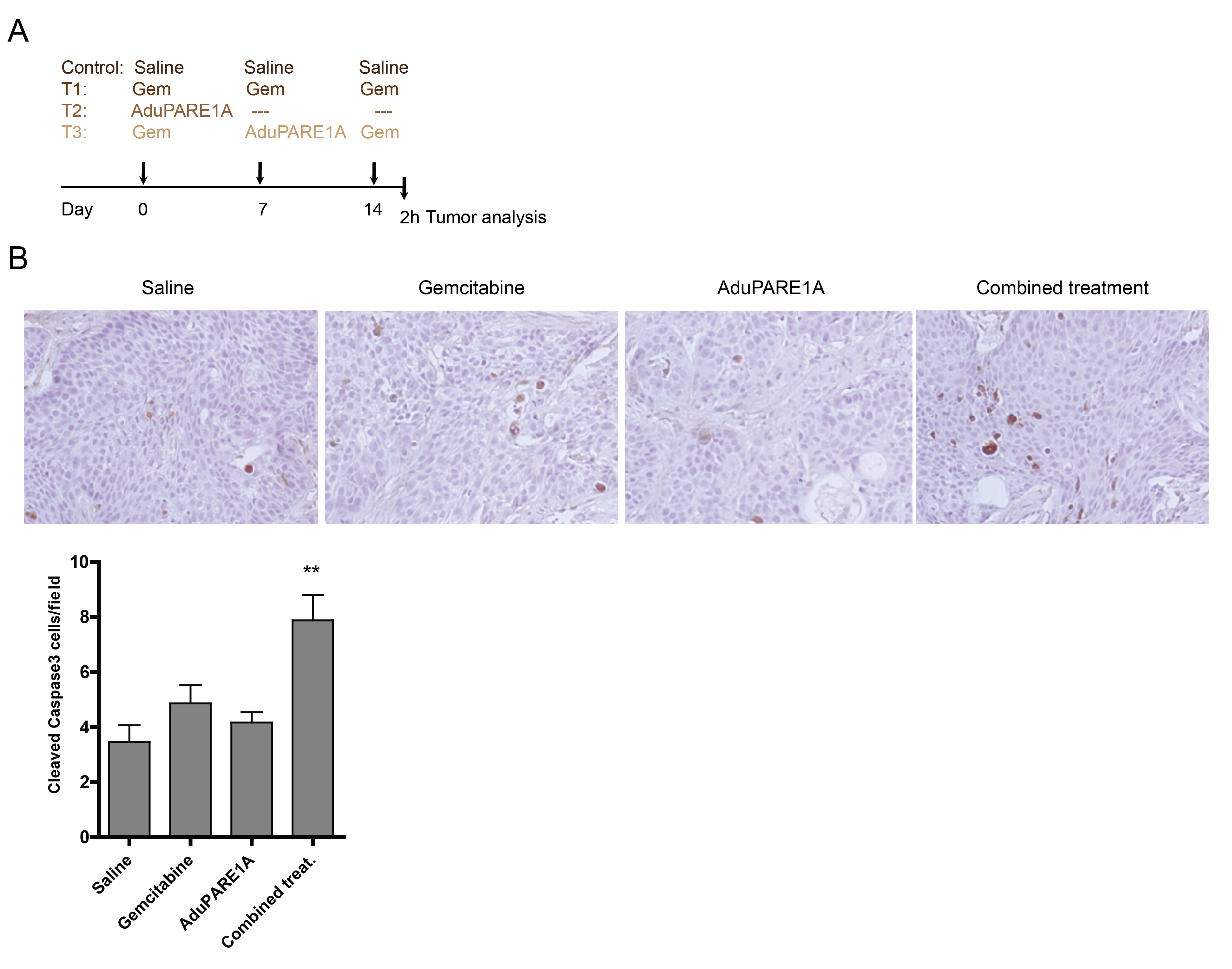

Supplement: Additional file 1: Figure S1. — A) Schematic representation of treatment protocols applied in mice bearing BxPC-3 subcutaneous tumors. B) Representative images of IHC staining for Cleaved-Caspase3 (Ref.9661, Cell Signaling Technology) in BxPC-3 tumors. Bar graph shows quantification of cleaved-caspase3 cells per field. Results are represented as the mean ± SEM of n ≥6 fields. (JPEG 110 kb) [file 12943_2015_413_MOESM1_ESM.jpg]

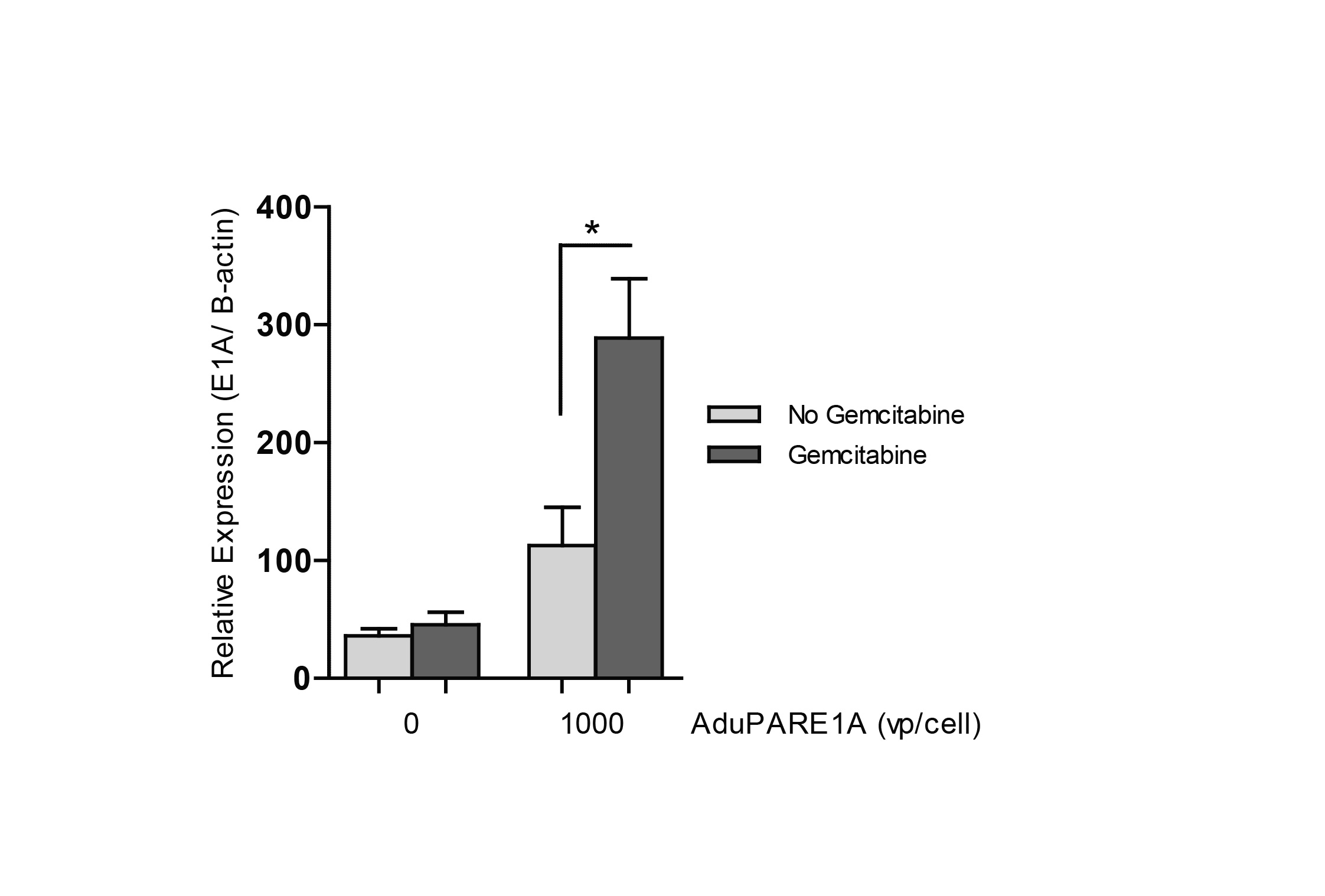

Supplement: Additional file 2: Figure S2. — Expression of E1A gene in 293T cells infected with AduPARE1A (1000 vp/cell) and treated, or not, with gemcitabine (200 ng/ml). Results represented are the mean ± SEM of at least four independent experiments. (DOCX 40 kb) [file 12943_2015_413_MOESM2_ESM.docx]
