# Supplementary material for: AduPARE1A and gemcitabine combined treatment trigger synergistic antitumor effects in pancreatic cancer through NF-κB mediated uPAR activation
Source: Mol Cancer. 2015 Jul 31;14:146. doi: 10.1186/s12943-015-0413-2 (PMC4521493; doi:10.1186/s12943-015-0413-2)
Supplement: Additional file 3: Table S1. — Primer sequences. (JPEG 1145 kb) [file 12943_2015_413_MOESM3_ESM.jpg]

**Supplementary Table S1.** Primer sequences

| GENES | PRIMER SEQUENCES | Amplicon Size |
| --- | --- | --- |
| uPAR | Fw 5’- gccttaccgaggttgtgtgt-3’ | 180bp |
|  | Rv 5’-catccaggcactgttcttca-3’ |  |
| E1A | Fw 5’- ATCGAAGAGGTACTGGCTGA -3’ | 405bp |
|  | Rv 5’- CCTCCGGTGATAATGACAAG-3’ |  |
| TK | Fw 5’- ATGGGGAAAACCACCACCAC-3’ | 196bp |
|  | Rv 5’- TGGGCGCTTGTCATTACCAC-3’ |  |
| Hexon | Fw 5’- GCCGCAGTGGTCTTACATGCACATC -3’ | 301bp |
|  | Rv 5’- CAGCACGCCGCGGATGTCAAAG -3’ |  |
| B-actin | Fw 5’- CTGGAACGGTGAAGGTGACA-3’ | 195bp |
|  | Rv 5’- GGGAGAGGACTGGGCCATT-3 |  |
